# More harm than good? Parental perspectives on dilatations in anorectal malformations

**DOI:** 10.1007/s00383-025-06070-2

**Published:** 2025-06-19

**Authors:** Ana Isabel Barros, Mário Rui Correia, Fátima Carvalho, Berta Bonet, Catarina Carvalho

**Affiliations:** Department of Pediatric Surgery of Centro Hospitalar Universitário de Santo António, Unidade Local de Saúde de Santo António, Porto, Portugal

**Keywords:** Anal dilations, Anoretal malformation, Caregiver perceptions, Posterior sagittal anorectoplasty, Psychological impact

## Abstract

**Purpose:**

Post-operative management for anorectal malformations (ARM) following surgical repair traditionally includes routine anal dilations (AD) to prevent anal strictures. Recent evidence questions its necessity and highlights psychological distress in patients and parents. We aimed to evaluate dilations’ psychosocial impact on both children and caregivers.

**Methods:**

Telephone questionnaire was performed to parents of ARM patients who underwent repair between 2009-2023, assessing their perceptions on AD and its’ child impact. Patient demographics, ARM classification, AD timing, and post-operative complications were collected.

**Results:**

Thirty-two caregivers participated. Median age at surgery was 5 months; at survey, 10.5 years. ARM with perineal fistula was the most common subtype. AD were performed preoperatively in 21.9%. Post-op, 15,6% did weekly calibrations, 62,5% daily AD. One-quarter of dilations/calibrations were performed by a surgeon in parents’ presence, of whom 62.5% reported emotional distress, 75% perception of child discomfort. Home-based AD parents reported difficulty in 54.2%, with two-thirds needing assistance, describing it as distressing for both themselves and their children. Overall, 28.1% perceived a negative psychological impact.

**Conclusions:**

Our findings highlight the significant psychological burden associated with AD. Given increasing evidence of its lack of benefit and psychological impact, our results support a careful re-evaluation of AD routine use.

## Introduction

Anal dilations (AD) following surgical correction of anorectal malformations (ARM) have been standard practice among most surgeons [1–3]. Although the rationale behind this practice is the prevention of stenosis following a circular anastomosis, increasing doubts have been raised about the actual effectiveness and necessity of routinely performing this painful procedure [1, 4]. The wide variability in dilation regimens described in the literature has fueled these concerns, as similar outcomes have been reported with very different approaches—ranging from daily home dilations to once-weekly sessions during surgical follow-up visits [1, 2, 5, 6].

Recently, two studies have indicated that AD’s impact may not be significant in preventing stenosis after PSARP (posterior sagittal anorectoplasty) [3, 4]. This finding has led to growing doubts about the true necessity and potential effects of this routine practice. Although AD is commonly performed, it remains an invasive and painful procedure [7]. In recent years, the psychosocial impact of AD on caregivers of children with Hirschsprung’s disease and ARM has been studied. Wehrli et al. found that more than half of caregivers experienced distress and reported emotional reactions, including guilt and stress, due to the procedure [1]. Additionally, another study established a correlation between AD and negative effects on both mental health and psychosocial interactions in adolescents after early age AD [8].

In light of these findings, we aimed to investigate parental perceptions of AD following PSARP for ARM surgical correction.

## Methods

For this retrospective study, a brief investigator-designed questionnaire (Table 1) was administered via telephone to parents of children who underwent ARM repair at our department, between 2009 and 2023.

**Table 1:** Questionnaire performed to parents of ARM patients

Question 1	Did you have difficulty performing the anal dilations? Yes or No?
Question 2	Did you need help from a second person to perform the anal dilations? Yes or No?
Question 3	Was there any discomfort for the baby during the anal dilations? Yes or No?
Question 4	Did you feel an emotional distress during the performance of anal dilations to your child? Yes or No?
Question 5	Do you feel that the anal dilations had a negative psychological impact on your child, i.e. was there any difficulty in carrying out physical exam or examination of perineal region? Yes or No?

### Anal dilations regimen

In our department, postoperative evaluation guides AD protocol. Fourteen days postoperatively, every patient is assessed during a consultation. If the anal caliber, measured with Hegar dilators, remains unchanged or decreases by only one size, outpatient calibrations with the same size Hegar probe are performed at each visit. However, if a decrease of two or more sizes is observed, suggesting anal stenosis, home-based AD using Hegar dilators is prescribed. Alternatively, if parents are unable or unwilling to perform home AD, these may be performed in the outpatient setting. Additionally, AD is routinely implemented in patients with perineal or vestibular fistulas as part of standard management.

### Survey

The survey comprised five dichotomous (yes/no) questions assessing parental perceptions of AD’s feasibility, its impact on the caregiver, and the child’s perceived discomfort and potential long-term psychological impact.

All caregivers who performed home AD before and/or after surgical repair were included in the study. Additionally, parents of children who underwent anal calibrations or AD performed by the surgeon in their presence were also included and responded to questions 3–5 of the questionnaire.

Adolescents were not included in this study due* to* the substantial time lapse between the period of anal dilations and the time of the survey, raising concerns about the reliability and accuracy of their recollections. Therefore, parental reports were considered a more appropriate and consistent source of information regarding early postoperative experiences.

### Data collection

Demographic and clinical data were retrieved from the patients’ medical records, which included age, sex, ARM subtype, past medical history, history of previous stoma, surgical technique, AD timing (preoperative, postoperative, or both), and postoperative complications.

### Statistical analysis

Descriptive statistics were used to analyze the data. Frequencies and percentages were calculated for categorical variables, while median and range were used for continuous variables.

To assess whether significant differences existed between the responses of patients who underwent AD versus calibrations, a comparative analysis was performed using the Chi-square test for independence. Fisher’s exact test was applied when any expected cell count was below 5 to ensure test validity.

Additionally, to explore potential correlations between caregiver responses and the type of anorectal malformation (grouped as perineal fistula, vestibular fistula, rectourethral fistula, no fistula, rectum-bladder neck fistula and cloacal malformation), as well as between caregiver responses and the presence or absence of postoperative complications, the same tests were conducted.

A p-value < 0.05 was considered statistically significant. All analyses were performed using SPSS Version 30.

## Results

During the analysis period, 39 patients with ARM underwent surgical correction, and all of their caregivers were contacted by telephone. Of these, 3 could not be reached and were excluded from the study. Additionally, 4 patients were excluded due to no use of AD or calibrations: two children with perineal fistulas who underwent early surgical repair within the first two days of life, one child with ARM without fistula who underwent repair at three years of age, and one child with a vestibular fistula who underwent repair at six months of age. After exclusion, 32 parents of children with ARM were included, 94% of whom were female.

### ARM patients demographics and characteristics

Of the included patients, 62.5% were male. The median age at ARM surgery was 5 months. At the time of the survey, the median age was 10.5 years.

All ARM subtypes were represented within the sample, with ARM with perineal fistula being the most common subtype (56%). Fifty-three percent of our patients underwent neonatal colostomy creation prior to definitive surgical correction. Regarding associated comorbidities, 53% had isolated ARMs. In the remaining cases, additional anomalies were present, most commonly genitourinary abnormalities (41%). Further details on patient demographics and clinical characteristics are presented in Table 2.

**Table 2:** Patients demographics and clinical characteristics

	Count/median	Percentage/range
Age at surgery (months)	5	(0–77)
Age at survey response (years)	10.5	(1–21)
Male gender (N)	20	62.5%
ARM subtype - female (N)		
ARM with perineal fistula	8	25%
Cloacal malformation	2	6.3%
ARM with vestibular fistula	1	3.1%
ARM with vaginal fistula	1	3.1%
ARM subtype - male (N)		
ARM with perineal fistula	10	31.2%
ARM with rectourethral fistula	5	15,6%
ARM without fistula	4	12.5%
ARM with rectum-bladder neck fistula	1	3.1%
History of previous stoma (N)	17	53.1%
Comorbidities (N)		
Genitourinary	13	40.6%
Cardiac	4	12.5%
Vertebral	3	9.4%
Limbs	2	6.3%
Mullerian anomalies	2	6.3%
Genetic syndrome	3	9.4%)

Postoperative complications occurred in 34% of patients, with anal suture dehiscence (n = 5) being the most common, followed by mucosal prolapse (n = 3), anal stenosis (n = 2), wound infection (n = 2), and anal misplacement (n = 1). Surgical reintervention was required in five cases.

### Dilatations and caregiver survey

Of the 32 patients assessed, 63% underwent ADs and 16% underwent calibrations during the postoperative period. Preoperative dilations were performed in 7 patients, exclusively in those with a perineal fistula, representing 44% of all cases with this malformation subtype.

#### Anal dilatations performed by caregivers

**Fig. 1:**
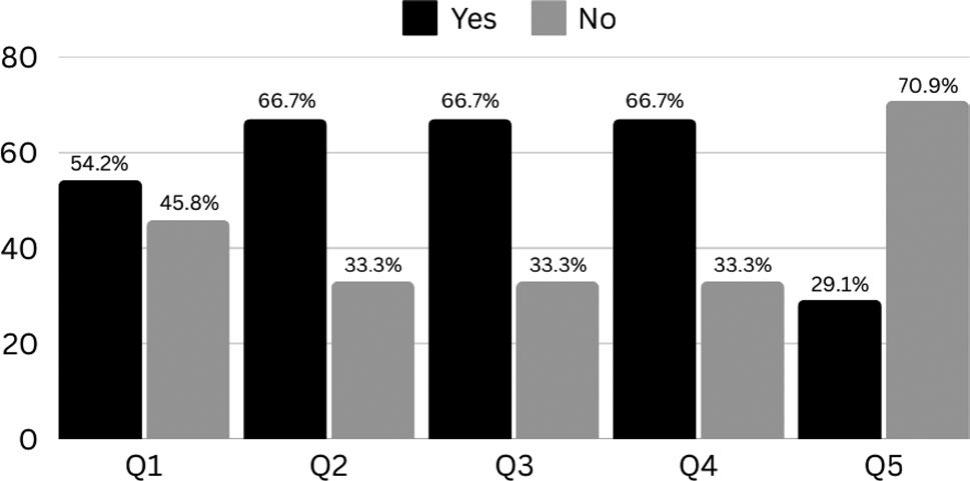
Survey answers about anal dilations performed by caregivers

Among caregivers who performed home dilatations (N = 24), 54% reported difficulties, with two-thirds requiring assistance from another person. Furthermore, two-thirds of caregivers found the procedure distressing for both themselves and their child (Fig. 1).

#### Anal dilatations/calibrations performed by surgeons

**Fig. 2:**
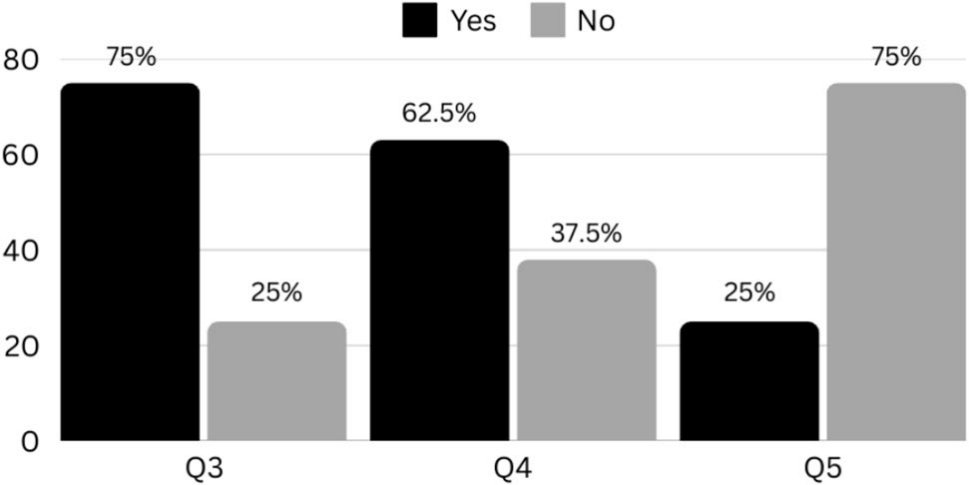
Survey answers about anal dilations/calibrations performed by surgeons

Twenty-five percent of all dilatations/calibrations were performed by a surgeon in the presence of the parents. Of these, 63% of parents reported experiencing emotional distress, and 75% perceived their child as experiencing discomfort during the procedure (Fig. 2).

In both groups, more than 25% reported a negative psychological impact after these maneuvers, specifically with difficulties in physical examination cooperation or resistance to perineal inspection upon physician evaluation.

Parental perception of discomfort varied between dilation and calibration procedures, and further details are presented in Table 3. No statistical differences were encountered in parents’ perception between AD and calibrations.

**Table 3:** Survey results for questions 3 to 5—comparison of caregivers’ perceptions between patients that underwent AD and those who underwent calibrations

	AD yes answer count (N; %)	Calibrations yes answer count (N; %)	*p*-value
Question 3	19 (70.4%)	3 (60%)	0,646
Question 4	19 (70,4%)	2 (40%)	0,209
Question 5	8 (29.6%)	1 (20%)	0,563

#### Analysis by ARM subtype and complications

No statistically significant associations were found between caregiver responses and the different ARM subtypes (question 3: p = 0.224; question 4: p = 0.481; question: 5 p = 1.000). Similarly, caregiver perceptions did not significantly differ between patients who experienced postoperative complications and those who did not ((question 3: p = 0.526; question 4: p = 0.582; question: 5 p = 0.638).

## Discussion

AD is an invasive and potentially painful intervention [7], and its clinical benefits, particularly in preventing stenosis, may be overshadowed by negative effects on the psychosocial development of patients and the emotional distress experienced by caregivers.

Our study’s aim was to evaluate our clinical experience with AD in ARM families, and to investigate the impact and perception of AD among our patient population and their caregivers.

Our findings revealed that most caregivers reported significant levels of distress and discomfort associated with AD, even when performed at home. More than half complained of difficulties performing the regimen, with most needing help from another person. However, the impact of AD appears to extend further, as approximately one-quarter of parents also noted possible lifelong consequences. Parents reported that after AD regimens, patient cooperation for physical examinations was negatively affected, particularly for perineal inspections or maneuvers.

These results highlight two significant concerns. The first is the immediate discomfort and fear associated with potentially harmful procedures in young children [7]. As we know, the emotional burden of surgery can be overwhelming for some parents, and patients with ARM often require multiple procedures at a very young age. Following definitive surgery, the need to perform another potentially painful, difficult, and repetitive maneuver, such as AD, adds further challenges, as evidenced in our population.

Secondly, it is noteworthy that such a procedure could potentially have long-term psychological effects on young children, as our findings suggest. Exaggerated fear and avoidance of physical examination are common and concerning in all children; however, in ARM patients, these issues present additional challenges. These patients often require prolonged surveillance and uncomfortable bowel management regimens, such as retrograde enemas [9]. If feelings of fear and resistance are introduced early in life, the potential for achieving good bowel control may be significantly compromised.

As pediatric surgeons, our mission is to minimize harm without compromising surgical outcomes. After analyzing the impact of AD on stenosis prevention and its consequences, we believe it is reasonable to reconsider the routine necessity of this potentially non-essential maneuver.

However, the use of AD may still be beneficial in selected cases. In these cases, we ought to provide all the necessary tools to minimize discomfort and long-lasting consequences, both logistically and emotionally. We strongly believe in the importance of well-structured programs where families can access comprehensive information and receive the necessary support to navigate the numerous procedures and surgeries their children may require [1, 10–12]. We recommend an initial consultation at the time of diagnosis, during which physicians and nurses thoroughly explain the key steps of ARM management—from birth through adulthood—helping families prepare for each stage of treatment. Additionally, a multidisciplinary team (surgeons, gastroenterologists, urologists, psychologists, nurses, among others) should be maintained in ARM care [10, 13]. Families may also benefit from sharing experiences, and patient groups and meetings should be encouraged [1, 13].

Despite the relevance of our findings, several limitations must be acknowledged. The retrospective design, based on parental recollection, introduces potential recall bias, particularly given the wide age range of participants. The small sample size and single-center setting also limit the generalizability of the results.

An additional limitation of this study is the exclusive reliance on parental reports, which may not fully reflect the child’s subjective experience. Although incorporating perspectives from older children could offer valuable insights, this approach is also susceptible to recall bias, particularly given that early childhood memories (as AD is normally performed within the first year of life) are often fragmented or poorly consolidated.

Moreover, the use of a non-validated, subjective questionnaire may affect the reliability of the data, with responses potentially influenced by social desirability or individual coping mechanisms. The absence of standardized tools to assess parental stress and child discomfort further limits the study’s external validity.

Future research is needed to overcome these limitations. Prospective, multicenter studies involving larger cohorts are necessary to validate these findings and evaluate ARM treatment options and long-term consequences. Incorporating standardized, validated psychometric tools for assessing parental stress and child discomfort would improve the objectivity and reliability of data collection.

## Conclusion

Routine AD remains widespread in the postoperative care for children with ARM, despite recent evidence questioning its necessity and potential long-term psychosocial consequences of this practice. Our findings reinforce these concerns, demonstrating significant distress among caregivers and perceived discomfort in children. We suggest a shift towards a more selective, individualized approach to AD. Care must be embedded within a multidisciplinary framework, prioritizing caregiver education, emotional support, and long-term follow-up, to improve outcomes and reduce long-term harm in this population.

## Data Availability

The data that support the findings of this study are available from the corresponding author upon reasonable request.
